# PGC-1α Induces Mitochondrial and Myokine Transcriptional Programs and Lipid Droplet and Glycogen Accumulation in Cultured Human Skeletal Muscle Cells

**DOI:** 10.1371/journal.pone.0029985

**Published:** 2012-01-17

**Authors:** Emma Mormeneo, Cecilia Jimenez-Mallebrera, Xavier Palomer, Valeria De Nigris, Manuel Vázquez-Carrera, Anna Orozco, Andrés Nascimento, Jaume Colomer, Carles Lerín, Anna M. Gómez-Foix

**Affiliations:** 1 CIBER de Diabetes y Enfermedades Metabólicas (CIBERDEM), Barcelona, Spain; 2 Departament de Bioquímica i Biologia Molecular, IBUB, Universitat de Barcelona (UB), Barcelona, Spain; 3 Unitat de Patologia Neuromuscular, Servei de Neurologia, Hospital Sant Joan de Déu, Barcelona, Spain; 4 Departament de Farmacologia i Química Terapèutica, IBUB, Universitat de Barcelona (UB), Barcelona, Spain; 5 Diabetes and Obesity Laboratory, Institute of Biomedical Research August Pi i Sunyer (IDIBAPS), Barcelona; Spain; Pennington Biomedical Research Center, United States of America

## Abstract

The transcriptional coactivator peroxisome proliferator-activated receptor-gamma coactivator 1 alpha (PGC-1α) is a chief activator of mitochondrial and metabolic programs and protects against atrophy in skeletal muscle (skm). Here we tested whether PGC-1α overexpression could restructure the transcriptome and metabolism of primary cultured human skm cells, which display a phenotype that resembles the atrophic phenotype. An oligonucleotide microarray analysis was used to reveal the effects of PGC-1α on the whole transcriptome. Fifty-three different genes showed altered expression in response to PGC-1α: 42 upregulated and 11 downregulated. The main gene ontologies (GO) associated with the upregulated genes were mitochondrial components and processes and this was linked with an increase in COX activity, an indicator of mitochondrial content. Furthermore, PGC-1α enhanced mitochondrial oxidation of palmitate and lactate to CO_2_, but not glucose oxidation. The other most significantly associated GOs for the upregulated genes were chemotaxis and cytokine activity, and several cytokines, including IL-8/CXCL8, CXCL6, CCL5 and CCL8, were within the most highly induced genes. Indeed, PGC-1α highly increased IL-8 cell protein content. The most upregulated gene was PVALB, which is related to calcium signaling. Potential metabolic regulators of fatty acid and glucose storage were among mainly regulated genes. The mRNA and protein level of FITM1/FIT1, which enhances the formation of lipid droplets, was raised by PGC-1α, while in oleate-incubated cells PGC-1α increased the number of smaller lipid droplets and modestly triglyceride levels, compared to controls. CALM1, the calcium-modulated δ subunit of phosphorylase kinase, was downregulated by PGC-1α, while glycogen phosphorylase was inactivated and glycogen storage was increased by PGC-1α. In conclusion, of the metabolic transcriptome deficiencies of cultured skm cells, PGC-1α rescued the expression of genes encoding mitochondrial proteins and FITM1. Several myokine genes, including IL-8 and CCL5, which are known to be constitutively expressed in human skm cells, were induced by PGC-1α.

## Introduction

The transcriptional coactivator PGC-1α, which regulates target genes through its interaction with diverse transcription factors and the recruitment of chromatin-remodelling complexes [1], [2], has been reported to play a major role in skm in both mitochondrial biogenesis and function [2] and metabolic programming [3], [4]; and in particular in metabolic adaptations to exercise [3], [4].

Induction of genes encoding mitochondrial proteins and mitochondrial biogenesis is one of the most powerful and consistent actions of PGC-1α in skm. Supporting data derive mostly from mouse studies. PGC-1α gene expression is enriched in skm type I (slow-twitch) fibers, which have a higher mitochondrial content and are more dependent on oxidative metabolism than type II (fast-twitch) fibers, which mainly use the glycolytic pathway [5]. Skm-specific transgenic overexpression of PGC-1α induces genes involved in mitochondrial electron transport [5], [6] and increases mitochondrial content [6]. Conversely, skm-specific PGC-1α knockout mice show decreased mitochondrial gene expression and function [7] or an attenuated exercise-induced rise in some mitochondrial electron transport chain proteins [8]. Furthermore, the expression of oxidative phosphorylation genes is blunted in skm of PGC-1α knockout mice [9]. It has been proposed that the powerful stimulation of mitochondrial function by PGC-1α is coordinately regulated with fiber type composition [5], [10], but not all data support this hypothesis [8], [9]. In transgenic mice, in which PGC-1α is controled by a promoter that is preferentially activated in type II fibers, induction of mitochondrial protein genes is linked to that of contractile protein genes enriched in type I fibers [5]. On the other hand, skm-specific PGC-1α knockout mice have a higher percentage of the glycolytic type IIx and IIb fibers at the apparent expense of the loss of oxidative type I and IIa fibers in different skm beds [10]. However, in another study using this type of murine model, endurance exercise-induction of IIb-to-IIa fiber type transformation was not attenuated by PGC-1α knockout [8]. Moreover, in PGC-1α knockout mice [9], no differences in fiber type composition were observed in the type I fiber-rich soleus muscle. Data on human skm are more limited. In one study, the amount of PGC-1α protein in different fiber types was found to follow the order: type IIa (fast oxidative-glycolytic)>type I (slow oxidative)>type IIx (fast glycolytic) fibers [11]. In another study [12], the percentage of type I fibers in human skm was positively correlated, and that of type IIa and type IIb (very fast glycolytic) fibers was negatively correlated, with PGC-1α mRNA.

PGC-1α orchestrates glucose and fatty acid metabolism in skm by regulating fatty acid and glucose utilization as fuel for oxidative phosphorylation. In this sense, PGC-1α enhanced the complete oxidation of fatty acids [13]–[15], while it inhibited glucose oxidation [13], [16] in cultured myotubes. Palmitate oxidation was also enhanced in subsarcolemmal mitochondria by PGC-1α overexpression in rat skm [17]. However, both strong stimulation of glucose uptake and slightly increased oxidation by PGC-1α was observed in C2C12 myotubes [18]. In addition, PGC-1α increases fuel stores despite the enhanced mitochondrial oxidative metabolism. In this regard, skm-specific PGC-1α overexpression in transgenic mice increased glucose uptake, glycogen and lipid droplet content [6]. In another transgenic murine model of skm-specific PGC-1α overexpression, higher intramyofibrillar lipid levels, including triglycerides, were detected [19]. In cultured human myotubes from lean and obese subjects, PGC-1α increased oleate incorporation into triglycerides [15].

PGC-1α is also involved in the maintenance of skm function and protection against damage. PGC-1α gene expression in skm is reduced in different conditions associated with atrophy, such as denervation in mice [20], spinal cord injury [12] and aging [21] in humans. Additionally, skm-specific knockout of the PGC-1α gene causes fiber damage and exercise intolerance in mice [10]. On the other hand, transgenic skm-specific PGC-1α overexpression reduces muscle wasting and the induction of atrophy-related genes due to denervation and fasting in mice [22]. Moreover, transgenic expression of PGC-1α in skm ameliorates a murine model of Duchenne muscular dystrophy (mdx) [23] and preserves mitochondrial function, neuromuscular junctions and muscle integrity during aging [24].

In this context, we aimed to establish whether PGC-1α overexpression could ameliorate the transcriptome and metabolism of primary cultured human skm cells, which display a reductive metabolic and muscle system transcriptome, compared to skm tissue, which resembles the atrophic phenotype [25]. Thus, we examined the effects of PGC-1α on the whole skm cell transcriptome and its possible relation to regulation of fatty acid and glucose metabolism. We show that PGC-1α activates the muscle cell transcriptional program of mitochondrial protein genes and mitochondrial biogenesis, whereas adult contractile protein genes are not regulated. We identify key metabolic control genes in fatty acid and glucose storage as new PGC-1α-target genes. Finally, a striking finding of this study is that PGC-1α activates a transcriptional program of myokines.

## Results

### Effects of PGC-1α on the cultured skm cell transcriptome

**Figure 1:**
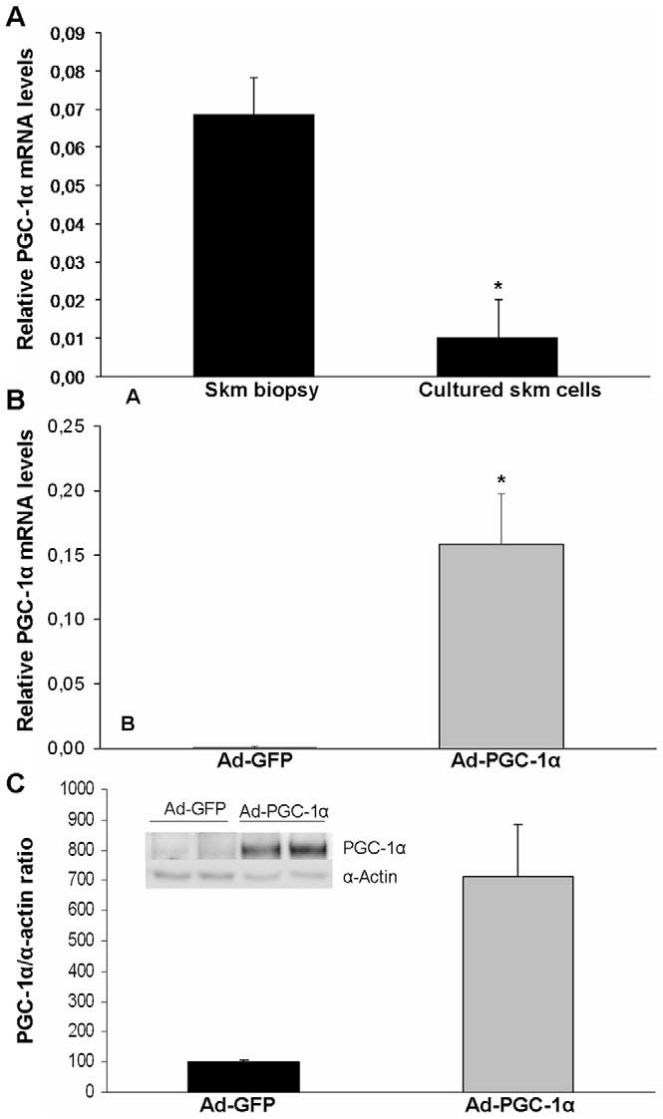
PGC-1α gene expression. (A,B) Human PGC-1α mRNA levels were determined relative to B2M using RT and real-time PCR. (A) Six samples were analyzed, three from cultured skm cells and three from skm biopsies. Data are expressed as mean values ± SEM of 2^−ΔCt^. The significance of the difference is *p<0.05. (B) Cultured skm cells were transduced with Ad-GFP or Ad-PGC-1α. Data are expressed as mean values ± SEM of 2^−ΔCt^ for three experiments performed in triplicate. The significance of the difference is *p<0.001. (C) Cultured skm cells were transduced with Ad-GFP or Ad-PGC-1α. Immunoblot analyses were performed on nuclear cell extracts (30 µg protein) and membranes were hybridized with antibodies against PGC-1α and α-actin. A representative image is shown. Bands were quantified with ImageQuant LAS 4000. Data are means ± SEM from two experiments performed in triplicate. Significance of differences versus cells treated with Ad-GFP: *p<0.05.

We first assessed whether PGC-1α transcript levels were altered in cultured skm cells compared to the biopsied paravertebral skm, composed of type I and type II fibers in similar proportions [26], [27], from which they were derived. We found that cultured skm cells had 16.4-fold lower PGC-1α mRNA levels than the skm biopsies (Figure 1A). We then overexpressed PGC-1α in the cultured skm cells using an adenovirus (Ad-PGC-1α) that encodes both mouse PGC-1α and EGFP proteins under the control of two separate CMV promoters, while control skm cells were treated with an adenovirus encoding EGFP (Ad-GFP). A probe for the mouse PGC-1α transcript revealed a gene expression level of 0.26±0.07 relative to the B2M gene in PGC-1α-cells, but no signal in control cells. A probe for human PGC-1α mRNA detected mouse PGC-1α mRNA in PGC-1α-cells (Figure 1B), at similar levels to the mouse-specific probe, thus showing that PGC-1α mRNA levels in PGC-1α-cells were close to those in the skm tissue. Immunoblotting analyses were carried out to quantify PGC-1α and GFP protein levels, the latter to control for adenoviral transduction load. PGC-1α protein content was increased by 7-fold in PGC-1α-cells compared to controls (Figure 1C). GFP analysis showed equivalent expression levels of the marker gene in controls and PGC-1α-cells (data not shown), indicating equivalent adenoviral transduction load.

An oligonucleotide microarray analysis was performed to reveal the effect of PGC-1α at the whole skm cell transcriptome level. Fifty-three different genes showed altered expression in cultured muscle cells overexpressing PGC-1α compared to control cells using the selection criteria of absolute fold change value >1.50 and p<0.025 (no significant differences were observed when using the corrected p-value). Of these, a majority (42 genes) were upregulated and only 11 were downregulated (Table 1).

**Table 1:** Differentially expressed genes in response to PGC-1α-overexpression in cultured muscle cells.

RefSeq	Gene symbol	Gene description	FC	P value
**Upregulated**				
NM_002854.2	PVALB	parvalbumin	+3.17	0.0049
NM_001825.1	CKMT2	creatine kinase, mitochondrial 2 (sarcomeric)	+3.04	0.0220
NM_000584.2	IL8	interleukin 8	+3.02	0.0108
NM_002993.2	CXCL6	chemokine (C-X-C motif) ligand 6 (granulocyte chemotactic protein 2)	+2.98	0.0068
NM_002985.2	CCL5	chemokine (C-C motif) ligand 5	+2.95	0.0017
NM_005952.2	MT1X	metallothionein 1X	+2.55	0.0034
NM_001024466.1	SOD2	superoxide dismutase 2, mitochondrial	+2.39	0.0003
NM_005623.2	CCL8	chemokine (C-C motif) ligand 8	+2.25	0.0001
NM_001151.2	SLC25A4	solute carrier family 25 (mitochondrial carrier; adenine nucleotide translocator), member 4	+2.25	8.89E-06
NM_001677.3	ATP1B1	ATPase, Na+/K+ transporting, beta 1 polypeptide	+1.93	0.0003
NM_002038.2	G1P3	Homo sapiens interferon, alpha-inducible protein (clone IFI-6-16) (G1P3)	+1.93	0.0194
NM_020529.1	NFKBIA	nuclear factor of kappa light polypeptide gene enhancer in B-cells inhibitor, alpha	+1.92	0.0002
NM_005954.2	MT3	metallothionein 3	+1.89	0.0014
NM_005530.2	IDH3A	isocitrate dehydrogenase 3 (NAD+) alpha	+1.86	0.0241
NM_001785.1	CDA	cytidine deaminase	+1.86	0.0159
NM_213720.1	CHCHD10	coiled-coil-helix-coiled-coil-helix domain containing 10	+1.80	0.0147
NM_032321.1	C2orf88	chromosome 2 open reading frame 88	+1.75	0.0101
NM_181679.1	NFS1	NFS1 nitrogen fixation 1 homolog (S. cerevisiae)	+1.73	0.0032
NM_022153.1	C10orf54	chromosome 10 open reading frame 54	+1.73	0.0002
NM_022440.1	MAL	mal, T-cell differentiation protein	+1.73	0.0036
NM_181755.1	HSD11B1	hydroxysteroid (11-beta) dehydrogenase 1	+1.72	0.0098
NR_001561.1	CYCS	cytochrome c, somatic	+1.71	0.0003
NM_006114.1	TOMM40	translocase of outer mitochondrial membrane 40 homolog (yeast)	+1.69	0.0002
NM_213650.1	SFXN4	sideroflexin 4	+1.69	0.0002
NM_001629.2	ALOX5AP	arachidonate 5-lipoxygenase-activating protein	+1.67	0.0027
NM_005950.1	MT1G	metallothionein 1G	+1.65	0.0184
NM_002982.3	CCL2	chemokine (C-C motif) ligand 2	+1.64	0.0110
NM_016098.1	BRP44L	brain protein 44-like	+1.64	0.0020
NM_003155.2	STC1	stanniocalcin 1	+1.63	0.0042
NM_032231.4	FAM96A	family with sequence similarity 96, member A	+1.63	0.0019
NM_003039.1	SLC2A5	solute carrier family 2 (facilitated glucose/fructose transporter), member 5	+1.62	0.0067
NM_198434.1	AURKA	aurora kinase A	+1.60	0.0010
NM_006528.2	TFPI2	tissue factor pathway inhibitor 2	+1.60	0.0066
NM_206963.1	RARRES1	retinoic acid receptor responder (tazarotene induced) 1	+1.60	0.0051
NM_004451.3	ESRRA	estrogen-related receptor alpha	+1.60	0.0001
NM_003000.1	SDHB	succinate dehydrogenase complex, subunit B, iron sulfur (Ip)	+1.59	0.0024
NM_001710.4	CFB	complement factor B	+1.57	0.0119
NM_203402.1	FITM1	fat storage-inducing transmembrane protein 1	+1.57	0.0016
NM_198594.1	C1QTNF1	C1q and tumor necrosis factor related protein 1	+1.57	0.0003
NM_014222.2	NDUFA8	NADH dehydrogenase (ubiquinone) 1 alpha subcomplex, 8, 19 kDa	+1.53	0.0003
NM_004753.4	DHRS3	dehydrogenase/reductase (SDR family) member 3	+1.53	0.0036
NM_001218.3	CA12	carbonic anhydrase XII	+1.51	0.0120
**Downregulated**				
NM_014059.1	C13orf15	chromosome 13 open reading frame 15	−1.73	0.0024
NM_002472.1	MYH8	myosin, heavy chain 8, skeletal muscle, perinatal	−1.64	0.0007
NM_016300.4	ARPP-21	cyclic AMP-regulated phosphoprotein, 21 kD	−1.64	0.0094
NM_006009.2	TUBA1A	tubulin, alpha 1a	−1.62	0.0002
NM_201525.1	GPR56	G protein-coupled receptor 56 (GPR56)	−1.61	0.0096
NM_032645.3	RAPSN	receptor-associated protein of the synapse	−1.59	0.0144
NM_001031692.1	LRRC17	leucine rich repeat containing 17	−1.56	0.0002
NM_006888.2	CALM1	calmodulin 1 (phosphorylase kinase, delta)	−1.53	0.0008
NM_000362.4	TIMP3	TIMP metallopeptidase inhibitor 3	−1.52	0.0008
NM_003068.3	SNAI2	snail homolog 2 (Drosophila)	−1.52	0.0036
NM_003246.2	THBS1	thrombospondin 1	−1.51	0.0003

Microarray analysis was performed on six cultured skm cell samples from three independent experiments, three transduced with Ad-GFP and three with Ad-PGC-1α adenovirus. The table lists the 53 regulated gene transcripts selected on the basis of the fold change (FC) and ordered by the magnitude of the absolute FC. Replicated genes, which occurred more than once in the microarray, were filtered: those appearing first in the list were arbitrarily chosen. The first column lists the NCBI Reference Sequence (RefSeq) accession number of the transcripts, the second the gene symbol, the third the description of the gene, the fourth the FC (with a positive symbol for upregulated genes and a negative symbol for downregulated ones) and the fifth the significance of differences as estimated by a paired t-test.

When GO tools were applied to the 53 differentially expressed genes in microarray (separately for upregulated and downregulated genes), and ontology selection criteria were restricted to an adjusted p value<0.01, only upregulated genes showed a significant association with GO (Table 2). With respect to the Cellular component, the main regulated GOs were related to mitochondria. Among the most regulated Biological processes were those involved in the generation of precursor metabolites and energy (including oxidation, cellular respiration and electron transport chain), those involving cytokines (such as wounding, locomotory behavior, inflammatory response, chemotaxis, taxis and leukocyte chemotaxis) and those participating in the removal or response to superoxide radicals. Remarkably, the most regulated Molecular function was the chemokine/cytokine-related function. Similar results were obtained when using KEGG (Kyoto Encyclopedia of Genes and Genomes) tools, i.e. the most regulated pathways were metabolic, chemokine and NOD-like receptor signaling pathways, cytokine-cytokine receptor interaction and Huntington's disease (data not shown).

**Table 2:** Gene ontology annotation of upregulated genes in response to PGC-1α-overexpression in cultured muscle cells.

	GENE ONTOLOGIES OF UPREGULATED GENES		
GO Term	GO term description	Number of genes	Adj p-value
	**Cellular component**		
GO:0005739	mitochondrion	12	0.00097
GO:0044429	mitochondrial part	10	0.00050
GO:0031975	envelope	10	0.00066
GO:0031967	organelle envelope	10	0.00069
GO:0005740	mitochondrial envelope	9	0.00029
GO:0031966	mitochondrial membrane	8	0.00090
GO:0005743	mitochondrial inner membrane	6	0.00429
GO:0019866	organelle inner membrane	6	0.00454
	**Biological process**		
GO:0042221	response to chemical stimulus	13	0.00105
GO:0065008	regulation of biological quality	13	0.00259
GO:0009605	response to external stimulus	9	0.00756
GO:0009611	response to wounding	8	0.00266
GO:0048878	chemical homeostasis	7	0.00741
GO:0007626	locomotory behavior	6	0.00364
GO:0006091	generation of precursor metabolites and energy	6	0.00422
GO:0006954	inflammatory response	6	0.00427
GO:0070887	cellular response to chemical stimulus	6	0.00443
GO:0006873	cellular ion homeostasis	6	0.00821
GO:0055082	cellular chemical homeostasis	6	0.00844
GO:0015980	energy derivation by oxidation of organic compounds	5	0.00158
GO:0006935	chemotaxis	5	0.00321
GO:0042330	taxis	5	0.00342
GO:0045333	cellular respiration	4	0.00333
GO:0022900	electron transport chain	4	0.00424
GO:0051260	protein homooligomerization	4	0.00424
GO:0001666	response to hypoxia	4	0.00965
GO:0030595	leukocyte chemotaxis	3	0.00428
GO:0060326	cell chemotaxis	3	0.00479
GO:0022904	respiratory electron transport chain	3	0.00826
GO:0050900	leukocyte migration	3	0.00965
GO:0048247	lymphocyte chemotaxis	2	0.00418
GO:0019430	removal of superoxide radicals	2	0.00432
GO:0071451	cellular response to superoxide	2	0.00447
GO:0071450	cellular response to oxygen radical	2	0.00464
GO:0000303	response to superoxide	2	0.00808
GO:0000305	response to oxygen radical	2	0.00933
	**Molecular function**		
GO:0008009	chemokine activity	5	0.00014
GO:0001664	G-protein-coupled receptor binding	5	0.00112
GO:0005126	cytokine receptor binding	5	0.00421
GO:0005125	cytokine activity	5	0.00486
GO:0046870	cadmium ion binding	2	0.00956

List of relevant GO annotated from the set of upregulated transcripts with an absolute FC>1.5 and p<0.01 in response to PGC-1α-overexpression. Only GOs with more than one gene observed are shown. The first column lists the GO term identifier, the second the GO term description, the third the number of genes that are annotated with this GO term, and the fourth the false discovery rate adjusted p value of that term.

**Figure 2:**
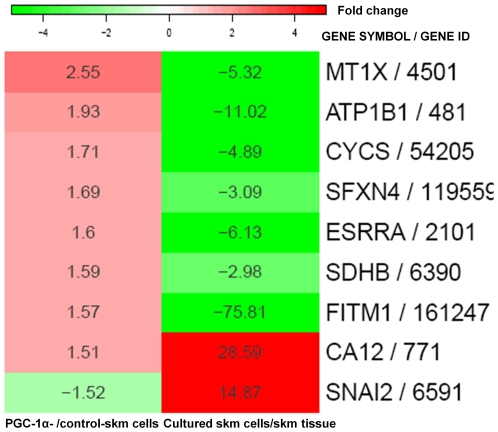
Genes regulated by both PGC-1α-overexpression in cultured skm cells and skm cultures in comparison to skm tissue. Heat map including the genes (Gene symbol/Gene ID for Entrez Gene) in Table 1 (PGC-1α- versus control-skm cells) that were also found to be differentially expressed after human skm culture according to Table S2 from [25] (cultured skm cells versus skm tissue). Each cell displays the absolute fold change in the corresponding comparison and is filled according to the color gradient shown at the top (green and red for down and upregulated genes, respectively).

To reveal the genes that were regulated in common in cultured skm cells by PGC-1α and in cultured skm cells compared to the skm biopsies, microarray data in this and our previous study [25] were analyzed together. As shown in Figure 2, nine genes were identified overall. All 7 genes that were downregulated by skm culture were induced by PGC-1α. Two of these genes (CYCS and SDHB) are involved in the electron transport system. Others participate in heavy metal binding (MT1X), iron transport (SFXN4), cation pumping (ATP1B) and the control of lipid metabolism (FITM1) [28]; and one was the estrogen-related receptor ESRRA. Of the two genes that were upregulated by skm culture, the transcription factor SNAI2, which triggers epithelial-mesenchymal transitions, was downregulated by PGC-1α, and the carbonic anhydrase CA12 was further upregulated PGC-1α.

The altered expression for 11 genes chosen from those identified in the oligonucleotide microarray was analyzed by reverse transcription (RT) and real-time PCR (Table 3). Altered expression was confirmed in all cases. The most upregulated gene in the microarray, PVALB, was one of these selected genes and the fold change measured by RT and real-time PCR was about 12, which was much higher than that measured in the microarray. Other genes were selected to represent the most regulated GOs, i.e. mitochondria (SLC25A4), chemokine activity (IL-8, CXCL6, CCL5 and CCL8) and an inhibitor of the NF-kappaB (NF-kB) complex (NFKBIA); and metabolic control (FITM1, SLC2A5, ARRPP-21 and CALM1). Among the selected upregulated genes, similar increases to those detected by the microarray were observed, except for FITM1, whose increment was higher in the RT and real-time PCR than in the microarray analysis. The two selected downregulated genes, ARPP-21 and CALM1, showed an absolute fold change value that was also higher according to RT and real-time PCR.

**Table 3:** Genes validated for differential expression in PGC-1α-overexpressing cultured muscle cells.

RefSeq	Gene symbol	Gene description	FC	P value
**Upregulated**				
NM_002854.2	PVALB	parvalbumin	+12.45	4.08E-8
NM_000584.2	IL8	interleukin 8	+2.81	0.03
NM_002993.2	CXCL6	chemokine (C-X-C motif) ligand 6 (granulocyte chemotactic protein 2)	+2.51	0.006
NM_002985.2	CCL5	chemokine (C-C motif) ligand 5	+4.21	0.0004
NM_005623.2	CCL8	chemokine (C-C motif) ligand 8	+2.64	0.001
NM_001151.2	SLC25A4	solute carrier family 25 (mitochondrial carrier; adenine nucleotide translocator), member 4	+2.91	0.0009
NM_020529.1	NFKBIA	nuclear factor of kappa light polypeptide gene enhancer in B-cells inhibitor, alpha	+1.39	0.04
NM_003039.1	SLC2A5	solute carrier family 2 (facilitated glucose/fructose transporter), member 5	+1.46	0.04
NM_203402.1	FITM1	fat storage-inducing transmembrane protein 1	+2.86	0.0008
**Downregulated**				
NM_016300.4	ARPP-21	cyclic AMP-regulated phosphoprotein, 21 kD	−2.04	0.05
NM_006888.2	CALM1	calmodulin 1 (phosphorylase kinase, delta)	−1.71	0.04

List of genes with changed expression levels in response to PGC-1α overexpression in cultured muscle cells as validated by RT and real-time PCR and ordered as in Table 1. For each gene, the FC in gene expression was calculated from the mean values in PGC-1α-cells versus control cells from six independent experiments performed in triplicate. FC has a positive symbol for upregulated genes and a negative symbol for downregulated genes. All columns are as in Table 1.

**Figure 3:**
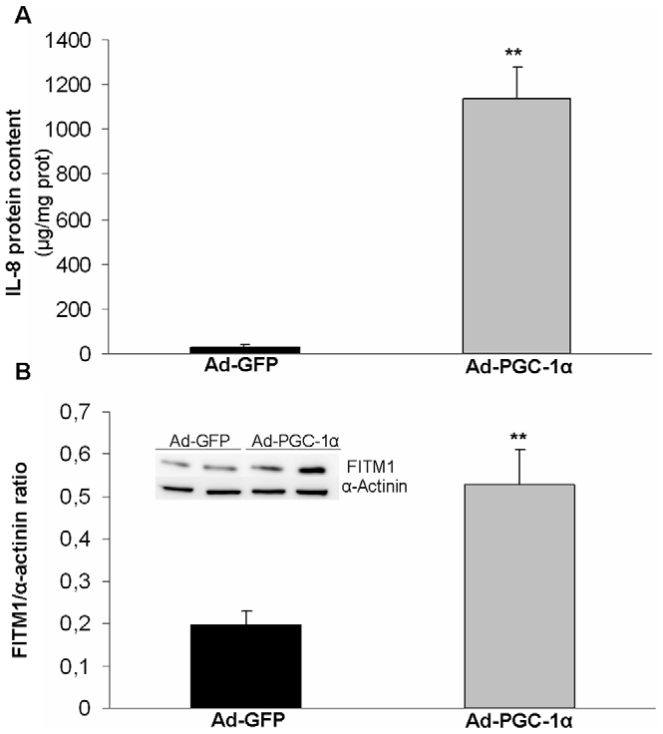
Upregulation of IL-8 and FITM1 protein content by PGC-1α-overexpression in cultured skm cells. Cultured skm cells were transduced with Ad-GFP or Ad-PGC-1α. In cell extracts, (A) ELISA assay for IL-8 and (B) immunoblot analysis of FITM1 were performed. (B) For FITM1, α-actinin was used as loading control; a representative image is shown and bands were quantified. (A and B) Data are means ± SEM from three experiments performed in triplicate. Significance of differences versus cells treated with Ad-GFP: *p<0.05.

The changed expression of two genes IL-8 and FITM1, was assessed at the protein level. IL-8 protein content in cell extracts from PGC-1α-cells was about 36-fold higher than in control cells (Figure 3A). IL-8 protein could not be detected in 48 h-conditioned media of neither PGC-1α- or control-cells, indicating that it is accumulated rather than secreted in cultured muscle cells. FITM1 protein content was 2.7-fold higher in PGC-1α-cells compared to controls (Figure 3B).

### Metabolic effects of PGC-1α overexpression in cultured muscle cells

**Figure 4:**
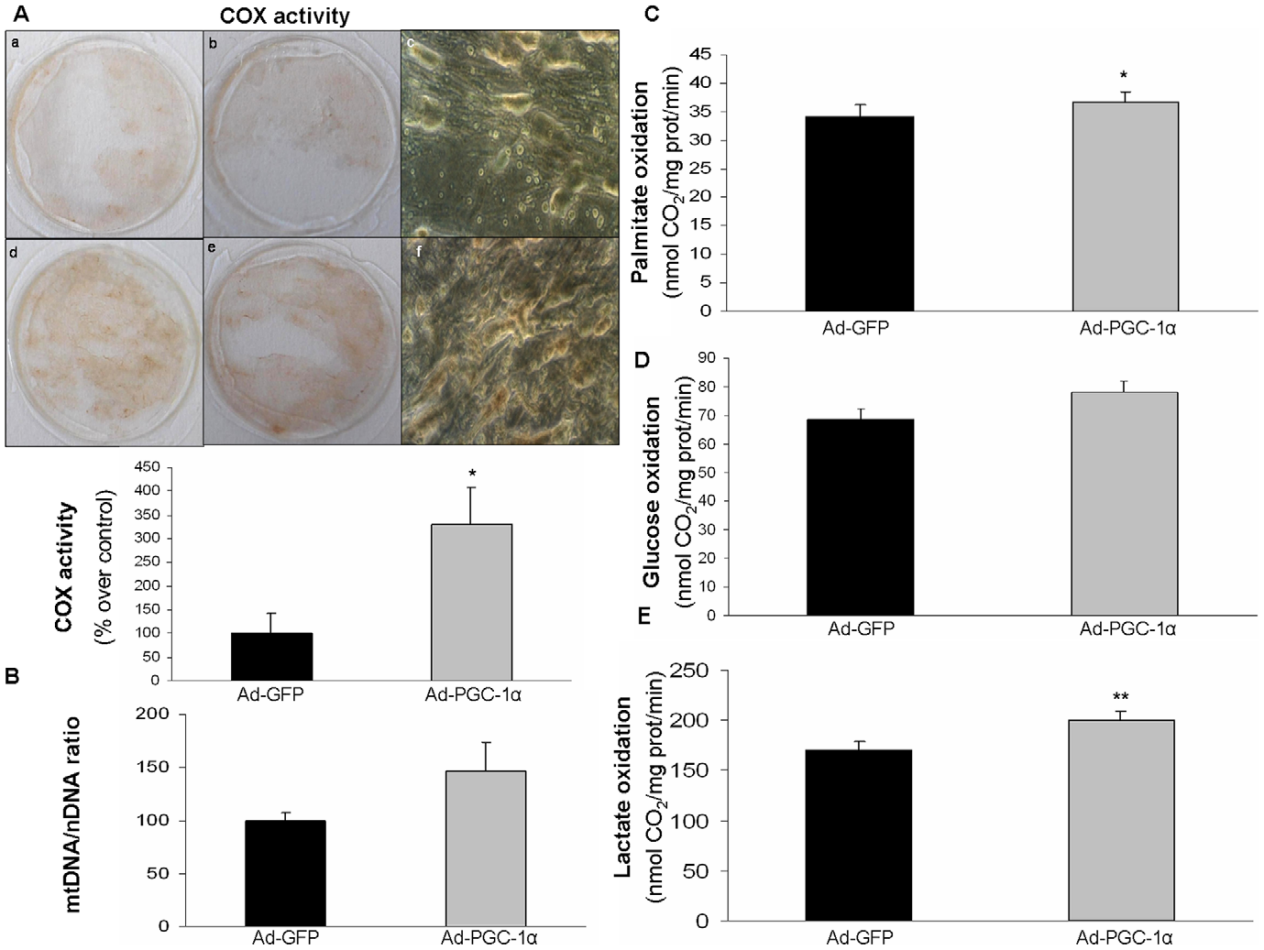
Effect of PGC-1α-overexpression on mitochondrial COX activity, mitochondrial to nuclear DNA ratio and oxidation of metabolic substrates in cultured muscle cells. Cultured muscle cells were transduced with Ad-GFP or Ad-PGC-1α adenoviruses. (A) Cytochemical staining of COX activity was performed in cultured skm cells that had been transduced with Ad-GFP (a, b and c) or Ad-PGC-1α (d, e and f). Images were obtained with a camera (a,b,d,e) or with an inverted microscope at 800× magnification (c,f). Images from two independent experiments performed in duplicate were quantitatively analyzed and the data expressed as percentages of the values in the controls: means ± SEM are shown on the graph. The significance of the difference is *p<0.005. (B) Cells were harvested and total DNA isolated to measure the ratio of mitochondrial DNA (mtDNA) content to nuclear DNA (nDNA) content. Data are expressed as a percentage of control. Data are means ± SEM from two experiments performed in triplicate. (C–E) Cells were then incubated for 4 h with: (C) 0.5 mM [1-^14^C]-palmitate (2.8 µCi/µmol), (D) 10 mM [U-^14^C]-glucose (0.21 µCi/µmol) and (E) 2 mM [U-^14^C]-lactate (0.6 µCi/µmol). Production of ^14^CO_2_ was subsequently quantified. Data are expressed as means ± SEM from two experiments performed in sextuplicate. The significance of the difference is *p<0.005 and **p<0.001.

To evaluate whether PGC-1α was regulating mitochondrial biogenesis in our cell model, we measured cytochrome c oxidase (COX) activity cytochemically and mitochondrial DNA content relative to nuclear DNA content. COX activity is a marker of mitochondrial content because it involves genes from the mitochondrial as well as nuclear genome. The intensity of COX activity staining was about 4-fold higher in cells overexpressing PGC-1α than in the controls (Figure 4A). In response to PGC-1α, a trend for higher mitochondrial to nuclear DNA ratio was observed, although this did not reach statistical significance (Figure 4B).

We then analyzed the mitochondrial oxidation of metabolic substrates to CO_2_. First, fatty acid oxidation was assessed by ^14^CO_2_ release from [1-^14^C]-palmitate (Figure 4C), revealing that it was enhanced by 13% in response to PGC-1α overexpression. In contrast, [U-^14^C]-glucose oxidation was unaltered by the forced expression of PGC-1α (Figure 4D), whereas [U-^14^C]-lactate oxidation was increased by 18% (Figure 4E). A PGC-1α inhibitory effect on glucose oxidation has been repeatedly reported for skm in association with the induction of pyruvate dehydrogenase kinase-4 (PDK4) gene expression [13], [16]. Thus, we measured PDK4 mRNA levels by RT and real-time PCR and found that they were increased in PGC-1α-overexpressing cells compared to controls (1.8-fold, p<0.001).

**Figure 5:**
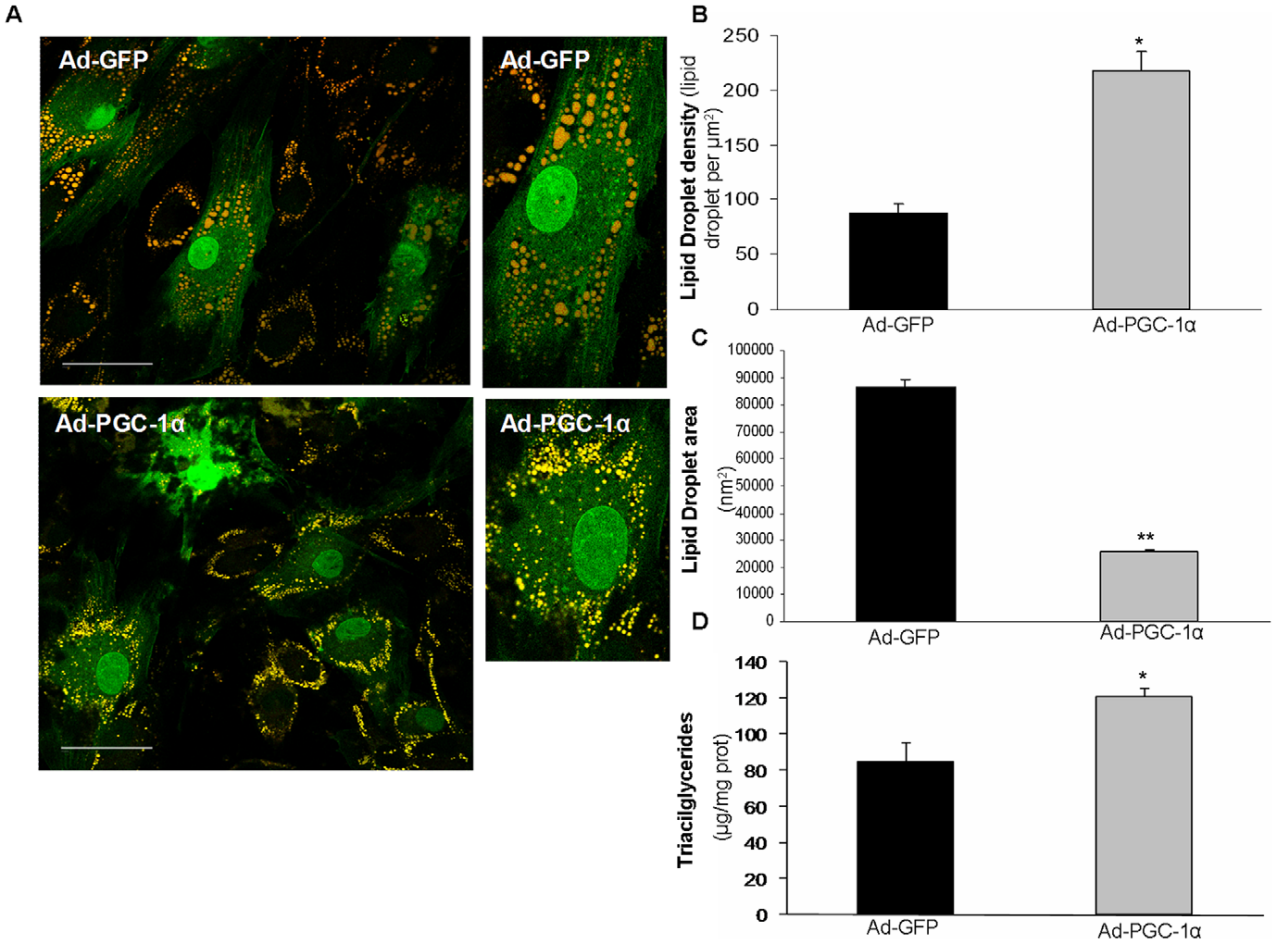
Effect of PGC-1α-overexpression on lipid droplets and triglyceride accumulation in cultured muscle cells. Cultured skm cells were transduced with Ad-GFP or Ad-PGC-1α and incubated with 0.5 mM oleate for 16 h. (A,B,C) Cells were fixed and then stained with Nile Red. (A) Representative lipid droplet micrographs obtained via confocal microscopy are shown. White bars represent 5 µm. (B) The number of lipid droplets per cell area and (C) the lipid droplet mean area values were quantified. Data are means ± SEM of (B) four cells or (C) at least 414 droplets. (D) Triglyceride content was measured. Data are means ± SEM of three experiments performed in quadruplicate. (B,C,D) The significance of the differences versus cells treated with Ad-GFP is *p<0.05 and **p<0.01.

We examined whether the lipid droplet content was modified in cultured skm cells treated with Ad-PGC-1α or Ad-GFP and incubated with oleate, a fatty acid that drives triglyceride synthesis (Figure 5A). In these conditions, PGC-1α overexpressing cells showed a 2.5-fold higher number of lipid droplets per cell (Figure 5B), but these droplets were smaller (70% lower mean area values) than controls (Figure 5C). The triglyceride content in cells incubated with oleate was increased by about 40% (Figure 5D). Since greater lipid accumulation by PGC-1α in skm has been associated with powerful upregulation of the FAT/CD36 gene in transgenic mice [5], [6], [19], [29], we measured its mRNA levels. We found that FAT/CD36 transcript levels, as assessed by RT and real-time PCR, were not modified in PGC-1α-overexpressing cells compared to controls (data not shown).

**Figure 6:**
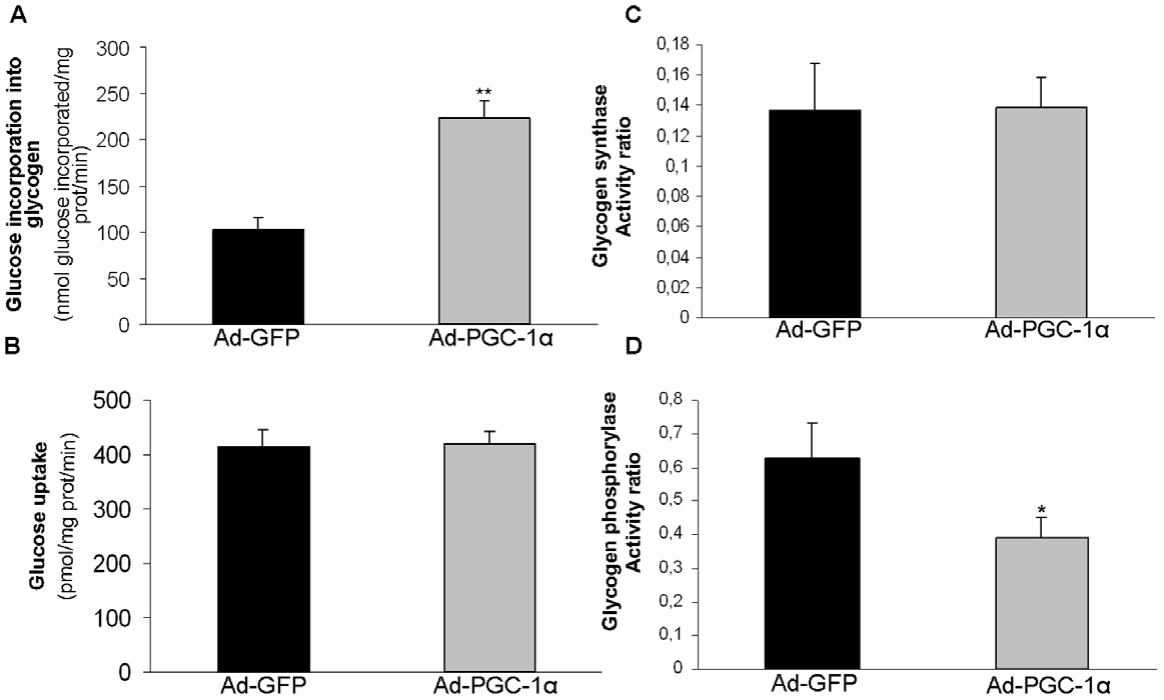
Effect of PGC-1α-overexpression on glycogen metabolism and glucose uptake in cultured muscle cells. Cultured skm cells were transduced with Ad-GFP or Ad-PGC-1α. (A) Cells were incubated with 10 mM [U-^14^C]-glucose (0.10 µCi/µmol) for 18 h and then harvested to asses glucose incorporation into glycogen. Data are means ± SEM from two experiments performed in triplicate. (B) Glucose uptake was measured using 0.5 mM 2-deoxy-D-[^3^H]glucose (0.5 µCi/well). Data are means ± SEM from two experiments performed in triplicate. (C,D) Cells were harvested to measure (C) glycogen synthase and (D) glycogen phosphorylase activity. Data are means ± SEM from three experiments performed in quadruplicate. The significance of differences versus cells treated with control adenoviruses is indicated as follows: *p<0.05 and **p<0.01.

Finally, we assessed glucose incorporation into glycogen. As shown in Figure 6A, this was markedly increased (about 2-fold) in response to PGC-1α. In contrast, glucose uptake was not (Figure 6B). To obtain more insight into the mechanism of action of PGC-1α in glycogen accumulation we measured the activity of glycogen synthase (Figure 6C) and glycogen phosphorylase (Figure 6D). The activity ratio of glycogen synthase (−/+glucose 6-phosphate) was unaffected by PGC-1α, as was total glycogen synthase activity (11.00±1.90 mU/mg protein in PGC-1α-cells and 9.22±1.62 mU/mg protein in control cells). The glycogen phosphorylase activity ratio (−/+AMP) was reduced by PGC-1α (37%), whereas total glycogen phosphorylase activity did not differ between the two groups (547±60 mU/mg protein in PGC-1α-cells versus 508±66 in controls). This reduced activation state of glycogen phosphorylase, together with no change in glycogen synthase activity, may explain the observed increase in the storage of glucose as glycogen in response to PGC-1α.

## Discussion

Cultured human skm cells, which display an atrophy-related transcriptome including metabolic and muscle system downregulation [25], are shown here to have decreased PGC-1α mRNA levels compared to the biopsied skm. This is in line with the observations of reduced PGC-1α gene expression in atrophy due to different basis including denervation [12], [20], which may account, at least in part, for the atrophic-related phenotype of the cultured skm cells.

The core effect of overexpressed PGC-1α on the cultured skm cell transcriptome, as revealed by an oligonucleotide microarray analysis, is the enrichment in the expression of genes related to mitochondrial components and processes, such as cellular respiration and the electron transport chain. This finding is consistent with other microarray analyses exploring the effects of PGC-1α at the whole skm cell transcriptome level [9], [14]. In rat L6 myotubes, adenovirus-mediated PGC-1α overexpression activated the expression of genes involved in the tricarboxylic acid, the electron transport chain and oxidative phosphorylation [14]. The expression of genes necessary for mitochondrial function was reduced in the skm of PGC-1α knockout mice, and these same genes were induced in C2C12 muscle cells with adenovirus-mediated overexpression of PGC-1α [9]. Moreover, several studies have reported the upregulation of diverse mitochondrial oxidative phosphorylation genes by PGC-1α in skm murine cells, as reviewed in [1], [3], [4]. According to our microarray data, the main mitochondrial protein PGC-1α target genes in human skm cells are the adenine nucleotide translocator SLC25A4/ANT1, the electron transporter CYCS and the oxidative stress resistance gene SOD2, all of which have been previously described as PGC-1α-induced genes [8], [9], [14], [24]. Our findings reinforce the notion that stimulation of the transcriptional mitochondrial program by PGC-1α does not require innervation, as previously observed in denervated skm of transgenic mice overexpressing PGC-1α [22]. Moreover, inspection of the genes that are regulated both by skm culture and PGC-1α-overexpression in cultured skm cells revealed that two downregulated mitochondrial protein genes were rescued by PGC-1α: CYCS and the beta subunit of the succinate dehydrogenase complex, SDHB, a component of the mitochondrial complex II. This demonstrates that PGC-1α can rescue the expression of some of the mitochondrial protein genes that are deficient in the cultured skm cells compared to the tissue from which they were derived and in this sense PGC-1α-cells transcriptome is more similar to that of skeletal muscle tissue.

PGC-1α stimulated mitochondrial biogenesis in our cultured skm cells, as indicated by the increase in cytochemical COX activity and the trend for higher mitochondrial to nuclear DNA ratio. The positive link between COX activity and PGC-1α gene expression has been established in tissues of varying oxidative capacity and in skm in response to contractile activity and thyroid hormone treatment in rats [30]. Furthermore, skm-specific transgenic expression of PGC-1α prevents the decline in COX activity during aging in mice [24] and the reduction in COX activity in a mouse model of mitochondrial myopathy caused by a skm-specific deletion of COX10 [31]. On the contrary, the reduction in COX activity is associated with the lack of PGC-1α in the skm tissue of the PGC-1α knockout mouse model [32]. We detected slight increases in the oxidation rate of palmitate and lactate in response to PGC-1α, in agreement with the observed rise in mitochondrial biogenesis, but no increase in glucose oxidation. As shown previously [13], [16], we found that this is associated with an increase in the mRNA levels of PDK4, the kinase that inactivates pyruvate dehydrogenase, which catalyses the oxidative decarboxylation of pyruvate to acetyl-CoA that can then enter the tricarboxylic acid cycle in the mitochondria.

PGC-1α-mediated induction of mitochondria related genes in cultured human skm cells was not accompanied by a switch in the fiber type-specific profile of contractile proteins. Only MYH8, which is an embryonic isoform, was among the most highly downregulated genes. This isoform is abundant in the cultured skm cells, which co-express fast-twitch adult (MYH2 and MYL1) and embryonic (MYH3, MYH8 and MYL4) skm myosin heavy (MYH) and light (MYL) chain isoforms at higher levels than slow-twitch adult isoforms (MYH7 and MYL3), as shown previously [33]. Our finding contrasts with the activation in skm-specific PGC-1α transgenic mice of contractile protein genes characteristic of type I fibers together with mitochondrial protein genes in putative type II muscles [5], or the increase in type IIx and IIb fibers in different skm beds in skm-specific PGC-1α knockout mice [10]. Our finding is instead consistent with a range of evidence that other signals influence the fiber phenotype besides PGC-1α. First, only a discrete number of type I and IIa fibers were distinguished in the plantaris muscle (type IIb fiber-rich) in skm-specific PGC-1α transgenic mice [5]. Moreover, the composition of fibers in the soleus muscle (type I fiber-rich) was not altered in PGC-1α knockout mice [9]. Additionally, there was no attenuation of the induction of IIb-to-IIa fiber-type transformation by endurance exercise in skm-specific PGC-1α knockout mice [8].

A number of genes that are highly regulated by PGC-1α in the cultured skm cells are involved in metabolic control and we provide an insight into the function of the potentially affected pathways. One of the upregulated genes was FITM1/FIT1, which was downregulated by skm tissue culture [25] and thereby rescued by PGC-1α. The FIT1 gene is abundantly expressed in mouse and human skm and its forced expression in cultured HEK293 cells strongly enhances the formation of lipid droplets with only a modest increase in triglyceride levels [28]. In line with this, we found that in muscle cells provided with oleate PGC-1α enhances the formation of lipid droplets, although these exhibit a lower mean area value, and moderately increases the triglyceride content. Noteworthy, in the brown fat cell line IMBAT-1, PGC-1α induced the gene encoding the cell death-inducing DFFA-like effector A (CIDEA), which in nonadipogenic cell lines promotes lipid droplet accumulation as well [34]. It has been consistently observed that transgenic overexpression of PGC-1α in skm increases lipid droplet and triglyceride accumulation, but that such an effect is linked to induction of the expression of the fatty acid translocase FAT/CD36 gene [6], [19]. FAT/CD36 enhances fatty acid uptake and triglyceride synthesis in cultured skm cells [35]. However, we found no upregulation of FAT/CD36 in response to PGC-1α in cultured skm cells.

Other PGC-1α-regulated genes related to metabolic control are the calmodulin CALM1 and its regulator ARPP-21, which are both downregulated. Calmodulins (CALM1, CALM2 and CALM3) are signal transducers that decode elevations in intracellular calcium [36], [37]. ARPP-21/RCS acts by sequestrating calmodulins, thereby preventing binding to its targets [38]. Calmodulins are the calcium-modulated δ subunit of the phosphorylase kinase enzyme, which is composed of four different subunits α, β, γ and δ. The γ subunit has catalytic activity and the others are regulatory subunits [39]. Phosphorylase kinase phosphorylates and activates glycogen phosphorylase, the rate-limiting enzyme of glycogenolysis. We show that glycogen phosphorylase is markedly inactivated in PGC-1α-cells and this is coupled to an increase in the storage of glucose as glycogen, whereas glycogen synthase activity is not affected. A stimulating effect of PGC-1α on glycogen storage has previously been reported in mice with skm-specific overexpression of PGC-1α, and this was associated with a reduced degree of phosphorylation of glycogen phosphorylase, suggesting inactivation of the enzyme as well [6]. However, in this transgenic mouse model the underlying mechanism was the reduction in the mRNA levels of phosphorylase kinase α subunit and glycogen phosphorylase. In our cell system, enhancement of glucose transport by PGC-1α, which could account for part of the increase in glycogen storage, was not observed. This is in contrast to the enhancement of glucose uptake by PGC-1α observed in isolated epitrochlearis muscle from skm-specific PGC-1α transgenic mice [6] and in other cultured muscle cells, such as L6 [40] and C2C12 [18] cells, in which forced expression of PGC-1α stimulated glucose uptake.

The gene that was most highly induced by PGC-1α in the cultured skm cells, PVALB, is also involved in calcium binding and signaling. PVALB protein is present mostly in rodent fast-twitch fibers and is involved in muscle relaxation after contraction [41]. PVALB has previously been identified as a main target gene of PGC-1α: in PGC-1α knockout mice, a marked reduction of PVALB mRNA levels was observed in the cerebrum, retina and heart; conversely a robust increase was detected after PGC-1α overexpression in neuroblastoma cells [42]. Remarkably, in that study, only a tendency for a reduction in PVALB gene expression was found in skm, probably because PGC-1α is mostly expressed in slow-twitch and PVALB in fast-twitch fibers. To summarize, the calcium signaling CALM1 and PVALB genes are targets of PGC-1α in muscle cells. In this regard, there is a well known relationship between calcium signaling and PGC-1α in skm, which involves the induction of the PGC-1α gene in response to cellular calcium influx [3], [4] and the action of PGC-1α as a downstream effector of the calcium/calcineurin pathway [3].

A striking finding in our oligonucleotide microarray analysis was the induction of a transcriptional cytokine gene program by PGC-1α. The GO analysis of upregulated genes revealed several Biological processes in which cytokines are involved and chemokine/cytokine activity as the most regulated Molecular function. Cytokine gene members of the CXC chemokine family [43], such as IL-8/CXCL8 and CXCL6 and CC chemokines, such as CCL5, CCL8 and CCL2, were among the most upregulated genes. Furthermore, skm cell IL-8 protein content was highly increased by PGC-1α, although no secretion of IL-8 into the media could be detected. Cytokines are known to be produced and secreted by skm (the so-called myokines), mainly in response to exercise, and exert either autocrine, paracrine or endocrine effects [44], [45]. In particular, IL-8 gene expression is greatly increased in contracting muscle, accompanied by only a small and transient net release in plasma, and has been proposed to participate in the exercise-induced angiogenesis of skm [46]. CCL5/RANTES is upregulated similarly in skm following acute resistance exercise in young and old men [47]. CCL2 is upregulated in idiopathic inflammatory myopathies and stimulates the proliferation of cultured myoblasts, as reviewed in [48]. Nevertheless, it is unclear whether some myokines are derived from infiltrated immune cells and result from an inflammatory reaction due to strenuous exercise [44]. Previous studies with cultured human myoblasts have shown, however, the constitutive gene expression of IL-1alpha, IL-6, IL-8 and RANTES [49], whereas IL-1beta, TNF-alpha [49] and CCL2 [48], [49] were detected only after treatment with pro-inflammatory stimuli. Our data conflict with the proposal that PGC-1α acts as a suppressor of the inflammatory response in skm [50]. The observations that support this concept come from skm-specific PGC-1α knockout mice, in which the expression of the inflammation marker genes TNF-alpha and IL-6 is increased in both skm and derived muscle cells in culture [7]; from skm-specific PGC-1α transgenic mice [24], which show reduced levels of TNF-alpha and IL-6; and adenoviral-mediated PGC-1α overexpression in C2C12 myotubes, which led to reduced TNF-alpha and IL-6 gene expression [7]. Remarkably, neither IL-6 nor TNF-alpha was detected as being regulated in the cultured skm cells in the current study. However, some of the myokines that were activated by PGC-1α, such as IL-8 and CCL5/RANTES, are known to be constitutively expressed by skm cells and upregulated by exercise, like PGC-1α [3], [4].

Finally, we observed slight upregulation of the gene encoding NFKBIA, which is an inhibitor of the NF-kB complex, by PGC-1α. NF-kB activates the expression of several inflammatory cytokine genes, including IL-8, CCL5/RANTES and CCL2/MCP1, and antioxidant enzyme genes [51]. NF-kB is activated in exercise and is considered to play a role in exercise-induced adaptations in skm, as PGC-1α is [52]. However, NF-κB activation downregulates PGC-1α [53]. Therefore, according to these data, we hypothesize that PGC-1α and NF-κB belong to alternative signaling pathways, which direct some skm exercise-associated effects, such as induction of myokine and antioxidant enzyme genes, while exerting a negative feedback signal on each other. On the other hand, activation of NF-kB-dependent transcription is also involved in several types of muscle atrophy [54]. Thereby, we suggest that by enhancing the NFKBIA pathway, PGC-1α could contribute to the prevention of muscle cell atrophy. For instance, none of the other genes that are known to be regulated by PGC-1α and involved in atrophy, such as atrogin-1 and MuRF-1 [22], were detected in the current microarray analysis.

In conclusion, PGC-1α activates the transcriptional program of mitochondrial protein genes and mitochondrial biogenesis in cultured human skm cells, as commonly observed in skm, and rescues the expression of some of the mitochondrial protein genes that are downregulated by tissue culture. Factors other than PGC-1α may be necessary to determine the muscle contractile protein fiber type. PGC-1α enhances lipid and glycogen storage in skm cells, while increasing the rate of fatty acid oxidation. The increased formation of lipid droplets may be linked to the upregulation of FITM1, and the enhanced glycogen accumulation to the inactivation of the glycogenolytic enzyme glycogen phosphorylase. Calcium binding proteins CALM1 and PVALB are PGC-1α targets in skm cells, adding to the evidence regarding its relationship with calcium signaling. A remarkable finding is that PGC-1α induces the expression of several myokines, providing new insights into the possible relationship between PGC-1α, inflammation and exercise.

## Materials and Methods

### Skeletal muscle cultures and adenoviral transduction

Myogenic cells and skm biopsies were obtained from a cryopreserved collection described in [25] that was obtained with written approval from the Ethics Committee of the Hospital Sant Joan de Déu according to Spanish legislation at that time. Written informed consent by patients devoid of neuromuscular disease was not sought since all samples were treated anonymously throughout the study. The ethics committee approved the waiver for consent for research. Cell differentiation was induced in confluent myoblast cultures; immediately after initiation of myoblast fusion, DMEM/M-199 medium (3∶1) with 10% FBS (fetal bovine serum), 10 µg/ml insulin, 4 mM glutamine, 25 ng/ml fibroblast growth factor and 10 ng/ml epidermal growth factor was replaced by DMEM/M-199 medium (3∶1) with 10% FBS and 10 µg/ml insulin, to further stimulate differentiation. Seven-days after induction of differentiation, cells were transduced with adenoviruses at a multiplicity of infection of 50 for 4 h. Cells were used 2 days after transduction, being depleted of insulin and FBS 24 h before the metabolic experiments. An adenovirus expressing EGFP (enhanced green fluorescent protein) under the control of the CMV (cytomegalovirus) promoter, Ad-GFP, was used as a control. The adenovirus expressing mouse PGC-1α cDNA (Ad-PGC-1α) constructed with the pAdTrack-CMV plasmid has been described elsewhere [55].

### Gene expression analysis

Total RNA was extracted from tissue and cell samples using the RNeasy minikit (Qiagen, Valencia, CA, USA) and homogenized using a Polytron (Kynematica Polytron, Westbury, NY, USA). RNA samples were quantified using the RiboGreen RNA Quantification Kit (Invitrogen, Paisley, UK), and monitored with the Agilent 2100 Bioanalyzer (Agilent Technologies, Santa Clara, CA, USA), to ensure high-quality RNA (RNA integrity ≥8).

Microarray analysis was performed in the Microarray Core Facility of the Centre de Regulació Genòmica (Barcelona, Spain) using the Human-6 v3.0 Expression BeadChip Kit-Array from Illumina. Briefly, biotinylated cRNA was prepared using the Illumina RNA Amplification Kit (Ambion, Austin, TX, USA) according to the manufacturer's instructions starting with approximately 200 ng total RNA. cRNA was purified and hybridized on Illumina Human-6 v3.0 Expression BeadChips following the manufacturer's instructions. After 16 h of hybridization arrays were washed, dried and scanned using Illumina BeadScan software on the Illumina BeadArray scanning system. Raw intensities were extracted using the GenomeStudio software (Illumina, San Diego, CA, USA). The data were normalized using the lumi package [56]. The microarray data are MIAME compliant and data files have been deposited in GEO Omnibus (access number GSE28206).

For RT and real-time PCR, an aliquot of 0.5 µg of total RNA was retro-transcribed with TaqMan RT reagents from Applied Biosystems (Foster City, CA, USA) using random hexamers and in a 20 µl total volume reaction. Real-time PCR was performed on 1 µl of the RT reaction mixture with the LightCycler 480 probes master and TaqMan Gene Expression Assays using the LightCycler 480 Real Time PCR System (Roche Diagnostics, Mannheim, Germany). Probes for SLC25A4 (Hs00154037_m1), SLC2A5 (Hs00161720_m1), FITM1 (Hs00416856_g1), PALB (Hs00161045_m1), ARPP-21 (Hs00372261_m1), CALM1 (Hs00237233_m1) and B2M (Hs99999907_m1) were obtained from Applied Biosystems. The Crossing Point (CP) values of the reference probe beta-2 microglobulin (B2M) were subtracted from the CP values of each target probe (ΔCP), and the ΔCP values of the control cells (transduced with AdGFP) were subtracted from the ΔCP values of PGC-1α- overexpressing cells. The data were expressed as mean values of (1/)2^−ΔCP^ or (1/)2^−ΔΔCP^. The significance of differences was estimated using a paired t-test.

In an alternative approach to RT and real-time PCR, Primer Express Software (Applied Biosystems, Foster City, USA) was used to design the forward (fp) and reverse (rp) primers: *18S* fp 5′-GCCGCTAGAGGTGAAATTCTTG-3′ and rp 5′-CATTCTTGGCAAATGCTTTCG-3′; *CCL5* fp 5′-CTGCATCTGCCTCCCCATA-3′ and rp 5′-GCGGGCAATGTAGGCAAA-3′; *CCL8* 5′-TGCTCATGGCAGCCACTTT-3′ and rp 5′-GCAGGTGATTGGAATGGAAACT-3′; *CXCL2* fp 5′-TGGGCAGAAAGCTTGTCTCA-3′ and rp 5′-TCAGCATCTTTTCGATGATTTTCTT-3; *CXCL6* fp 5′-CAGAGCTGCGTTGCACTTGT-3′ and rp 5′-ACACCTGCAGTTTACCAATCGTT-3′; *IκBα* fp 5′-CCCAAGCACCCGGATACAG-3′ and rp 5′-TCACTCTCTGGCAGCATCTGA-3′; *IL-6* fp 5′-CCCCCAGGAGAAGATTCCAA-3′ and rp 5′-TCAATTCGTTCTGAAGAGGTGAGT-3′; *IL-8* fp 5′-CTGGCCGTGGCTCTCTTG-3′ and rp 5′-TTAGCACTCCTTGGCAAAACTG-3′. The PCR reaction contained 10 ng of reverse-transcribed RNA and 10 µl of 2× iQ SYBR Green Supermix (Bio-Rad Laboratories, Barcelona, Spain) plus 900 nM of each primer. The optimal primer amplification efficiency for each primer set was assessed and a dissociation protocol was carried out to ensure a single PCR product. PCR assays were performed on a MiniOpticon Real-Time PCR Detection System (Bio-Rad). Thermal cycling conditions were as follows: activation of Taq DNA polymerase at 95°C for 10 min, followed by 40 cycles of amplification at 95°C for 15 s and 60°C for 1 min. All analyses were performed in duplicate and relative RNA levels were determined using 18S as the internal control.

### Statistical analysis and filtering and bioinformatic analysis

This analysis was performed in the Serveis de Suport a la Recerca of the Universitat de Barcelona. Statistical analysis was carried out using Bioconductor [57]. The lumi package [56], [58] was used to apply a log2 transformation followed by quantile normalization to the BeadStudio summarized data. Then, a paired t-test was performed using Limma [59] to select genes that were differentially expressed in PGC-1α-overexpressers and controls. Only those genes with a p value smaller than 0.025 and a fold change greater than 1.5 were selected (Table 1). GO analysis of differentially upregulated and downregulated genes was carried out as described in [60].

### Immunological methods

Cells were homogenized in buffer A consisting of 50 mM Tris-HCl (pH 7.5), 150 mM NaCl, 1 mM EDTA, 1 mM phenylmethylsulfonyl fluoride, 1 mM NaF, 1 mM Na_3_VO_4_, 2 mg/ml benzamidine, 2 mg/ml leupeptin, 1% (v/v) Nonidet P40, and 1 mM dithiothreitol. Lysates were then gently rocked for 60 min at 4°C, and stored at −80°C until analysis. Alternatively, to prepare cell nuclear extracts, cells were homogenized in buffer B consisting of 10 mM Tris/HCl (pH 7.0) 150 mM KF, 15 mM EDTA, 600 mM sucrose, 15 mM 2-mercaptoethanol, 17 µg/l leupeptin, 1 mM benzamidine and 1 mM PMSF. Homogenization of extracts was performed with the aid of a syringe 22 G. Homogenates were centrifuged at 1100× *g* for 10 min at 4°C and the pellet was collected. This nuclear enriched pellet was resuspended in the buffer A described above. Protein was resolved in 10% SDS-PAGE. Antibodies against GFP (8371-2, BD biosciences, Palo Alto, USA), PGC-1α (ST1202, Merck, Madrid, Spain), FITM1 (HPA019842, Sigma-Aldrich Madrid, Spain), α-actin (A2066, Sigma-Aldrich) and α-actinin (CBL231, Chemicon International, Temecula, CA, USA), were used. Horseradish peroxidase-conjugated secondary antibodies were used and membranes were developed with ECL-Plus (GE Healthcare, Buckinghamshire, UK). Protein bands were revealed and quantified using a LAS-3000 luminescent image analyzer (FujiFilm, Tokyo, Japan) or ImageQuant LAS 4000 (GE Healthcare Life Sciences, Uppsala, Sweden).

The cell extract for ELISA analysis was prepared by scraping cell monolayers with buffer B. Cell extracts were then sonicated and added with 1% Triton. The human IL-8 ELISA kit (Thermo Scientific, Waltham, MA, USA) and 50 µl of cell extracts were used.

### Cytochemistry

For cytochemical staining of COX activity, living cells seeded on glass coverslips were rinsed with PBS, air-dried for 1 h at room temperature and preincubated with 1 mM CoCl_2_, 10% sucrose in Tris-HCl (pH 7.6) for 15 min at room temperature. After rinsing with phosphate buffer (0.1 M sodium phosphate (pH 7.6) with 10% sucrose), the cells were incubated in the same buffer including 1 mg/ml cytochrome c, 1 mg/ml DAB (3-3′ diaminobenzidine-tetrahydrochloride dihydrate) and 0.1 mg/ml catalase for 6 h at 37°C. DAB, a compound that replaces complex III in the respiratory chain as an electron source and becomes a brown precipitate when it is oxidized by cytochrome c. The coverslips were rinsed once with the phosphate buffer and then PBS and distilled water. Cells were then fixed with PBS containing 4% (w/v) paraformaldehyde for 20 min, rinsed with PBS and mounted using Immuno Floure Mounting Medium. Coverslips were examined under an Olympus LH50A microscope and images were taken and then analyzed using the Multi Gauge software 3.0.

To stain for intracellular lipid droplets we used a fluorescent hydrophobic probe. Muscle cells seeded on glass coverslips were fixed as described above. Paraformaldehyde was aspirated off and the cells were rinsed with PBS. Nile red dissolved in DMSO to form a 1 mM stock solution was added in PBS (at 1∶1000 dilution) and incubated for 5 min. This was then aspirated and the cells rinsed with PBS. Coverslips were mounted with Immuno Floure Mounting Medium. Fluorescence images were obtained using a Leica TCS SP2 microscope. The size and number of lipid droplets were measured using AnalySIS (Soft Imaging System, Olympus, Germany).

### Metabolic analysis

We used [U-^14^C]-glucose to detect the incorporation of radioactivity into glycogen. We used [U-^14^C]-glucose, [1-^14^C]-palmitate or [U-^14^C]-lactate to detect oxidation to CO_2_ as previously described [61], except that for CO_2_ measurement cells were grown in 24-well plates. [U-^14^C]-glucose incorporation into glycogen, glycogen synthase and glycogen phosphorylase activities was measured as described in [61]. 2-Deoxy-D-glucose uptake was measured as described in [62]. Sodium salts of palmitic and oleic acids were prepared in deionized water containing 1.2 equivalents of NaOH at 70°C, until an optically clear dispersion was obtained. The fatty acid salt solution was added to DMEM containing fatty acid-free bovine serum albumin (BSA) with continuous agitation at a fatty acid: BSA molar ratio of 5∶1. To determine the triglyceride content extracts were prepared by scraping cell monolayers into a buffer consisting of 50 mM Tris (pH 7.9), 100 mM KCl, 20 mM KF, 0.5 mM EDTA and 0.05% Lubrol PX and applying three 5 s pulses of sonication. Homogenates were centrifuged at 11.000 *g* for 15 min and the resulting supernatants were collected. Protein concentration was measured using the Bio-Rad protein assay reagent. TAGs were measured enzymatically using a kit from Biosystems (Barcelona, Spain).

To determine the mitochondrial DNA content relative to the nuclear DNA content, total DNA was isolated from cells using the TRI Reagent (Sigma-Aldrich) and following manufacturer's protocol. Real-time PCR was carried out with the LightCycler system using SYBR® Green JumpStart™ Taq Ready Mix (Sigma-Aldrich). Primers for mitochondrial cytochrome c oxidase I, COX1, were: fp 5′-CCCCTGCCATAACCCAATACC A-3′; rp 5′-CCAGCAGCTAGGACTGGGAGAGA-3′. Primers for nuclear myogenin (myogenic factor 4), MYOG, were: 5′-AGGTGCTGTCAGGAAGCAAGGA-3′; antisense 5′-TAGGGGGAGGAGGGAACAAGGA-3′. The Ct values for the COX1 and MYOG probes were obtained and the ratio calculated.
